# Early Life Stress Increases Metabolic Risk, HPA Axis Reactivity, and Depressive-Like Behavior When Combined with Postweaning Social Isolation in Rats

**DOI:** 10.1371/journal.pone.0162665

**Published:** 2016-09-09

**Authors:** Javier Vargas, Mariana Junco, Carlos Gomez, Naima Lajud

**Affiliations:** División de Neurociencias, Centro de Investigación Biomédica de Michoacán–Instituto Mexicano del Seguro Social, Morelia, Michoacán, México; Brock University, CANADA

## Abstract

Early-life stress is associated with depression and metabolic abnormalities that increase the risk of cardiovascular disease and diabetes. Such associations could be due to increased glucocorticoid levels. Periodic maternal separation in the neonate and rearing in social isolation are potent stressors that increase hypothalamus-pituitary-adrenal axis activity. Moreover, social isolation promotes feed intake and body weight gain in rats subjected to periodic maternal separation; however, its effects on metabolic risks have not been described. In the present study, we evaluated whether periodic maternal separation, social isolation rearing, and a combination of these two stressors (periodic maternal separation + social isolation rearing) impair glucose homeostasis and its relation to the hypothalamus-pituitary-adrenal axis and depressive-like behavior. Periodic maternal separation increased basal corticosterone levels, induced a passive coping strategy in the forced swimming test, and was associated with a mild (24%) increase in fasting glucose, insulin resistance, and dyslipidemia. Rearing in social isolation increased stress reactivity in comparison to both controls and in combination with periodic maternal separation, without affecting the coping strategy associated with the forced swimming test. However, social isolation also increased body weight gain, fasting glucose (120%), and insulin levels in rats subjected to periodic maternal separation. Correlation analyses showed that stress-induced effects on coping strategy on the forced swimming test (but not on metabolic risk markers) are associated with basal corticosterone levels. These findings suggest that maternal separation and postweaning social isolation affect stress and metabolic vulnerability differentially and that early-life stress-related effects on metabolism are not directly dependent on glucocorticoid levels. In conclusion, our study supports the cumulative stress hypothesis, which suggests that metabolic risk markers arise when vulnerable individuals are exposed to social challenges later in life.

## Introduction

The prevalence of obesity has rapidly increased over the past two decades and represents a major public health concern. Obesity, hyperlipidemia, hypertension, and diabetes mellitus type 2 are common comorbidities in patients with depression. Nevertheless, genetic associations between cardiometabolic risk and mood disorders account for a small proportion of the incidence of these diseases [1]. The pathogenesis of metabolic syndrome (MetS) and its relation to depression is complex and poorly understood; however, the interactions between chronic stress, hypercortisolism, and psychotrauma are considered as contributing factors to the development of those disorders [2]. Patients with a history of depression have a higher risk of developing type 2 diabetes and this association cannot be entirely attributed to the use of antidepressant drugs or obesity [3]. In addition, recent evidence suggests that impaired insulin sensitivity in women with major depression can be improved after successful treatment with antidepressants [4]. Altogether, these findings suggest that a common etiology could increase the risk of both conditions, rather than depression being the sole complication of a metabolic imbalance in the brain.

There is consistent epidemiological evidence that links adverse fetal environments to an elevated risk of MetS and depression; however, the effect of childhood stress on metabolic risk is poorly understood. Early environmental influences, such as child abuse and neglect [5,6], social stress [7,8], and socioeconomic position [9,10] are emerging as contributors to the development of both depression and metabolic disease [11]. In addition, early-life stress (ELS) is associated with increased vulnerability to psychiatric disorders [12] and impaired cognitive performance [13]. The adaptive changes made by developing organisms in response to environmental stimuli result in permanent changes in physiology, structure, and metabolism, a phenomenon referred to as *early-life programming*. ELS programs hypothalamus-pituitary-adrenal (HPA) axis activity and increases basal glucocorticoid (GC) levels [14,15]. As GCs have a strong effect on neuronal survival, energy metabolism, and glucose homeostasis, elevated GC levels have been implicated as a major mechanism of stress-related adult pathologies, such as MetS and depression [2,16,17]. Moreover, increased GC secretion associated with ELS might contribute to adult phenotypic variability. Thus, it is likely that at least in vulnerable individuals, depression and metabolic risk could both be consequences of early-life programming of HPA axis reactivity [18,19].

Periodic maternal separation (MS180) is a widely used rodent model of an adverse early-life experience that consists of separating the pups from their mothers for periods of 3 h a day during the first weeks of life [20–22]. MS180 in adult males leads to increased basal corticosterone (CORT) levels, a passive coping strategy on the forced swimming test (FST) [22], decreased hippocampal neurogenesis, and impaired cognitive performance [23]. In addition, maternal separation from postnatal day 10 (P10) to P15 can influence the fate of adipose tissue proliferation, presumably leading to obesity later in life [24]. Postweaning social isolation rearing (SIR) is a potent stressor that promotes obesity in rats that have been subjected to MS180 [25]. Early disruption of social interactions, such as SIR, produces profound endocrine and behavioral effects [26,27]. SIR increases basal GC levels and HPA axis reactivity [28], and augments immobility in the FST [29]. MS180 and SIR reportedly do not cause obesity when presented separately [28,30]; however, SIR promotes feed intake and weight gain in MS180 rats [31], and induces sustained hyperphagia when MS180 animals are subjected to a fasting/re-feeding cycle. Such behavior has been proposed as a binge-like eating disorder [32].

The cumulative stress hypothesis suggests that adult pathologies could arise if vulnerable individuals are exposed to further challenges in later life. Although the afore mentioned studies demonstrate an interaction between ELS-induced vulnerability and SIR on weight gain and obesity, they do not evaluate neuroendocrine and metabolic phenotypes through functional studies. Therefore, the aim of the present study was to test the hypothesis that ELS increases metabolic risk in animals that are subjected to an additional social stressor. To test this, we evaluated HPA axis reactivity in rats subjected to MS180 that were housed under normal and SIR conditions, and correlated the results with depresive-like behavior and metabolic risk parameters.

## Materials and Methods

### Animals

**Fig 1:**
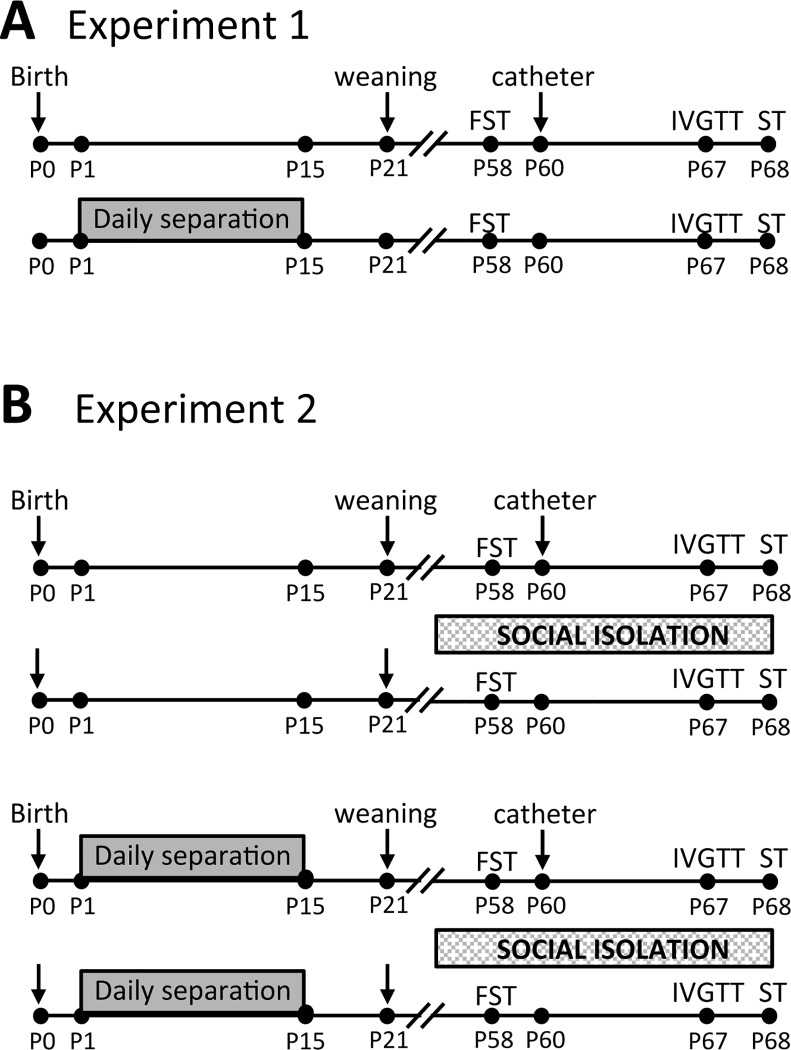
Experimental design. A) To characterize the effect of maternal separation (MS180) on metabolic risk and its relation to HPA axis activity, pups were weaned at P21 and group housed until adulthood. B) In order to evaluate whether social isolation rearing (SIR) increases MS180-related effects, half the animals were randomly assigned to SIR (single housing) or standard group housing (AFR) at weaning. All animals were subjected to the forced swimming test (FST) at postnatal day 58 (P58). All groups were catheterized on P60 and subjected to the intravenous glucose tolerance test (IVGTT) on P67. HPA axis stress responsivity after a 2 min cold swim stress (ST) was evaluated on P68.

Male Sprague-Dawley rats, 60–67 d of age, with a body weight range of 283–323g were used for all experiments. Female Sprague-Dawley rats were obtained from our institutional animal facility and mated in our facilities at approximately 10 weeks of age. Animals were maintained in standard temperature-controlled rooms with a 12-h light/dark cycle (lights on at 07:00 h), with free access to food and water (5008 Formulab Diet for breeding and 5001 Laboratory Rodent Diet for maintenance; Purina, LabDiet, St. Louis, MO, USA). The date of birth was designated as postnatal day zero (P0). On P1, pups from all same-age litters were cross-fostered and randomly assigned to each dam, litters were culled to eight pups (four to six males and two to four females) and each dam and litter were randomly assigned to the periodic maternal separation protocol (MS180), or left undisturbed with their mothers except for routine cage cleaning once a week (control, or CONT). Since early life handling has been shown to program HPA axis reactivity, manipulation of the CONT group was kept to a minimum throughout all procedures, and adequate nesting material (paper towers/sawdust) was provided. To characterize the effects of MS180 on metabolic parameters and its relation to HPA axis activity (experiment 1; Fig 1), pups were weaned at P21 and group housed (two to three siblings per cage) until adulthood (animal facility reared, AFR). To evaluate further whether SIR increases metabolic risk in animals subjected to MS180 (experiment 2), a different group of animals were used, and half of the animals were randomly assigned to single housing (SIR) or standard group housing (AFR) at weaning. Rats remained undisturbed until adulthood. To avoid sex- and litter-dependent effects, only males were evaluated, and each experimental group included animals from three to four different dams. All animals were tested in the FST at the age of 2 months and implanted with a jugular vein catheter. Animals recovered for 1 week after surgery and were then subjected to the intravenous glucose tolerance test (IVGTT). The catheters were closed and rats were allowed to recover for 24 h before HPA axis stress responsivity was evaluated (Fig 1).

### Methods to minimize potential pain and distress

All procedures and samplings were in concordance with the recommendations of the “Metabolic Phenotyping Guidelines for Assessing Glucose Homeostasis in Rodent Models” [33]. All experiments were carried out in accordance with the Official Regulations for Use and Care of Laboratory Animals of Mexico (NOM-062-ZOO-1999), the General Law of Health of Mexico, and the Institute of Laboratory Animal Resources’ *Guide for the Care and Use of Laboratory Animals* (National Research Council, revised 1996). In accordance with these regulations, all animals were euthanized with a sodium-pentobarbital overdose (50 mg/kg) at the end of the study.

All efforts were made to minimize animal suffering and reduce the number of animals used. Trained and skilled animal care personnel carried out all procedures, to prevent potential pain or distress to the animals. Neonatal manipulations were carried out under strict control measures, noise was kept to a minimum, and all personnel were provided with appropriate protective garments (latex gloves) to avoid the transfer of foreign odors to the nest. Surgical procedures were performed under ketamine/xylazine anesthesia by a skilled surgeon, in order to minimize tissue trauma and post-operative pain. Post-operative temperature was maintained at 32°C until recovery from anesthesia. After all surgical operations, animals were left to recover for one week in individual cages and handled daily to minimize distress. The Institutional Animal Care and Use Committee (Comité Local de Investigación en Salud No 1602) specifically approved the procedures of the present study.

### Periodic maternal separation

At 09:00 h, dams were placed in a separate cage with clean bedding, and provided with food and water, while the litter was removed. Pups were isolated in a small poly (methyl methacrylate) (Plexiglas®) cage with clean sawdust (novel environment), and separated from each of their siblings by cardboard. Separated pups were kept apart from the main colony and relocated to an adjacent room, where they were placed on a heating pad set at 30–32°C for 180 min (MS180). At the end of this period, the pups were returned to the original home-cage in the main colony room and the dam was returned to the nest. The entire procedure was repeated daily from P1 to P14.

### Forced swimming test

The FST was conducted as described previously [22,34]. Adult rats were placed individually into a Plexiglas® cylinder(35× 50 cm) filled with water to a depth of 40 cm, at 21°C for 10 min. Behavior was recorded with the use of a webcam. Struggling behavior consisted of upward-directed movements of the forepaws. Swimming behavior consisted of horizontal movements within the cylinder/swim chamber. Immobility was defined as a lack of paw movements with the animal floating. The data were collected by an experimenter blinded to the conditions.

### Jugular vein catheterization and blood sampling

On P60, animals were chronically implanted with intravenous catheters into the jugular vein that facilitated both infusion and regular blood sample collection in conscious unrestrained rats, without the need for handling. Animals were implanted with a 15-cm Silastic®/polyethylene (Dow Corning Corporation, MI, USA) catheter under ketamine (80mg/kg) / xylazine (6mg/kg) anesthesia, as previously described [22,35]. Post-operative temperature was maintained until recovery from anesthesia by placing the animals in a heating pad set at 32°C. After surgery, the rats were individually housed in Plexiglas® cages and left to recover for 1 week. During this time, animals were handled daily to avoid any effects of manipulation. Following recovery, rats were fasted overnight and then subjected to the IVGTT. The following day, they were tested for HPA axis reactivity. These two tests were separated by one day to facilitate recovery of the animals. For blood sampling, the catheter was attached to an extension tube (polyethylene; outside diameter, 1.0 mm) connected to a syringe filled with sterile heparinized saline (20 IU/mL) at 09:00 h, and the rats were left undisturbed for 120 min. Blood samples were withdrawn (0.2mL) and immediately substituted by sterile 0.9% saline. Baseline IVGTT (overnight fasting) blood samples were taken before intravenous infusion of 1 g/kg glucose (50%, DX-50; Pisa, Guadalajara, Mexico) and further samples were taken at 15, 30, 60, and 120 min after administration. To evaluate HPA axis reactivity, samples were first taken under basal stress-free conditions, and then animals were subjected to a 2-min cold swim stress with the extension tube of the intravenous catheter still attached. Further blood samples were taken at 5, 15, and 60 min after the application of stress. All blood samples were collected in tubes containing EDTA (5% solution, 5 μL/200 μL blood) supplemented with aprotinin (0.039 trypsin inhibitor units/tube; Sigma, St. Louis, MO, USA), and then centrifuged at 4000 rpm at 4°C. Plasma samples were stored at -30°C until further analysis.

### Metabolic phenotyping and endocrine parameters

Blood glucose concentration was measured using an Accu-Check glucose meter (Roche Diagnostics, Edo. Mexico, Mexico). The hormone content of the basal samples was assessed in duplicate using commercially available ELISA kits for insulin (Crystal Chem, Downers Grove, IL, USA; sensitivity, 0.156 ng/mL; intra-assay coefficient of variability, 5.8%) and corticosterone (Enzo Life Sciences, Farmingdale, NY, USA; sensitivity, 26.99 pg/mL; intra-assay coefficient of variability, 4.8%). Colorimetric assays were performed for triglycerides and low-/high-density lipoprotein cholesterol (Randox, London, UK). The quantitative insulin sensitivity check index (QUICKY) was calculated according to the formula 1/(log(insulin) + log(glucose)) [33]. To convert insulin from ng/mL to pmol/L, we multiplied by 150 [36].

### Statistical analysis

All statistical analyses were performed using the statistical software GB-stat V6.0 (Dynamic Microsystems, Silver Spring, MD, USA). Statistical significance was assessed by standard two-tailed Student’s *t*-tests and ANOVA with Fisher’s posthoc corrections, as appropriate. Stress reactivity and the IVGTT were analyzed using repeated measures ANOVA. For experiment 2, data were compared by a two-way ANOVA, with MS180 and rearing conditions as factors. Body weight gain, stress reactivity, and IVGTT data were compared using repeated measures three-way ANOVA, with time as the repeated factor, and MS180 and rearing conditions as fixed factors. If the ANOVA revealed a statistical difference, the Fisher’s post hoc test was used to determine group differences. ANOVA summary tables are presented as supporting information (S1 Table). Models with and without litter included as factors were compared to assess the degree to which mother-infant interactions influenced the outcome variables; however, these effects were negligible for all evaluations.

Simple linear regression analyses were performed to investigate correlations. Within-group measures of fasting CORT and metabolic indices, and forced swimming stress-induced CORT levels and behavior parameters were correlated using the Pearson’s product-moment correlation. Any given test with a probability (p) value <0.05 was considered significant. Only animals with complete sets of data were included in the analysis. An ANCOVA model was used to estimate the association between HPA axis basal activity and metabolic indices. In these models, “fasting CORT” or “stress-induced CORT” were used as continuous predictors and included as covariates, and fasting plasma glucose, insulin, body weight, and behavior were response variables. Group treatment was used as fixed categorical factors. A significant effect of a particular treatment indicated that the effect of the response variables persisted, even after controlling for CORT levels; therefore, no CORT associations were noted under the various experimental conditions.

## Results

### MS180 induced a passive coping strategy in the FST and increased basal HPA axis activity

**Fig 2:**
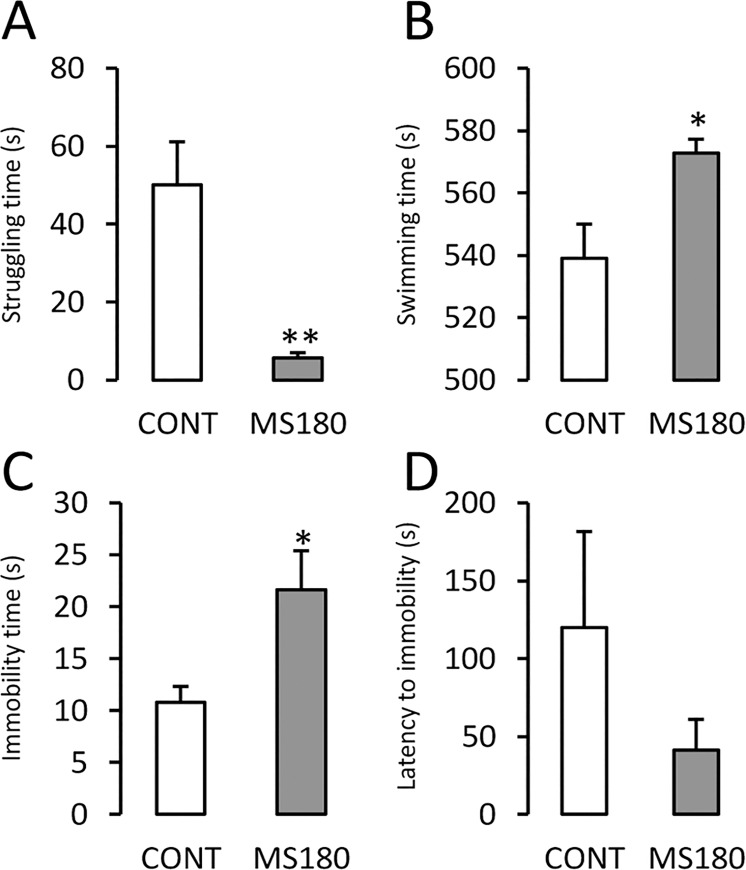
Maternal separation induces a passive coping strategy in the forced swimming test. A) Time spent struggling; B) time spent swimming; C) immobility time; and D) latency to immobility of maternally separated (MS180) and control (CONT) adult males. (Mean ± SEM, Student’s *t*-test *p ≤ 0.05, **p ≤ 0.01; n = 8)

**Fig 3:**
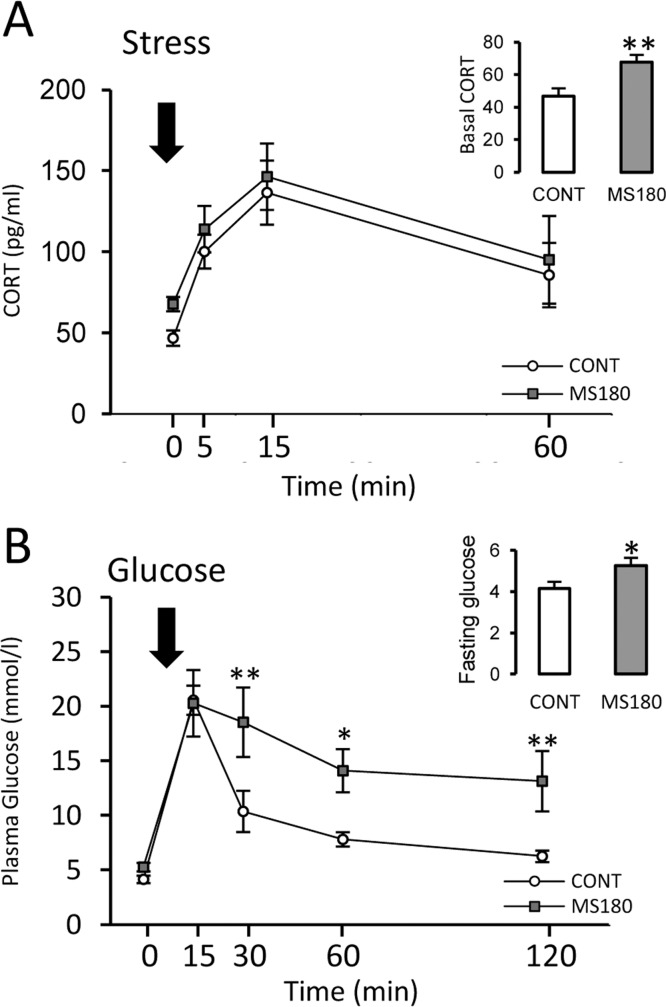
Maternal separation increases basal HPA axis activity and impairs glucose homeostasis. A) Corticosterone (CORT) plasma levels of maternally separated (MS180) and control (CONT) adult males before (inset) and at 5, 15, 30, and 60 min following stress exposure (2-min cold swim stress). B) Plasma glucose levels in the IVGTT after a 12h fast (inset) and at 15, 30, 60, and 120 min after an intravenous load of glucose (50%, 1mg/kg). (Mean ± SEM, ANOVA*p ≤ 0.05, **p ≤ 0.01; n = 8)

In the FST (Fig 2), maternal separation decreased struggling time (p ≤ 0.005,) and increased the time spent swimming (p ≤ 0.05) and immobility time (p ≤ 0.05) when compared to the CONT males. Maternally separated animals exhibited increased basal activity of the HPA axis in adulthood (Fig 3A). MS180 animals showed a 44.8% increase in CORT (Fig 3A, inset; p ≤ 0.01); however, repeated measures ANOVA only showed a significant effect of time after stress exposure (F_3,63_ = 12.3, p < 0.0001). No significant effects of treatment were observed.

### MS180 affected glucose homeostasis

In the IVGTT, glucose administration in MS180 rats caused a significantly greater increase in blood glucose levels, at 30 and 60 min post-injection, as compared to the controls (Fig 3B). Statistical analysis revealed significant effects of maternal separation (F_1,79_ = 9.1, p = 0.009) and time after glucose administration (F_1,79_ = 20.5, p < 0.0001), without further interactions. Moreover, the glucose values in MS180 rats failed to be restored after 120 min (p ≤ 0.001 vs. basal). Furthermore, MS180 rats presented a 24% increase in fasting glucose levels (Fig 3B, inset; p ≤ 0.05) when compared to the controls.

### SIR increased stress reactivity without further behavioral effects

**Fig 4:**
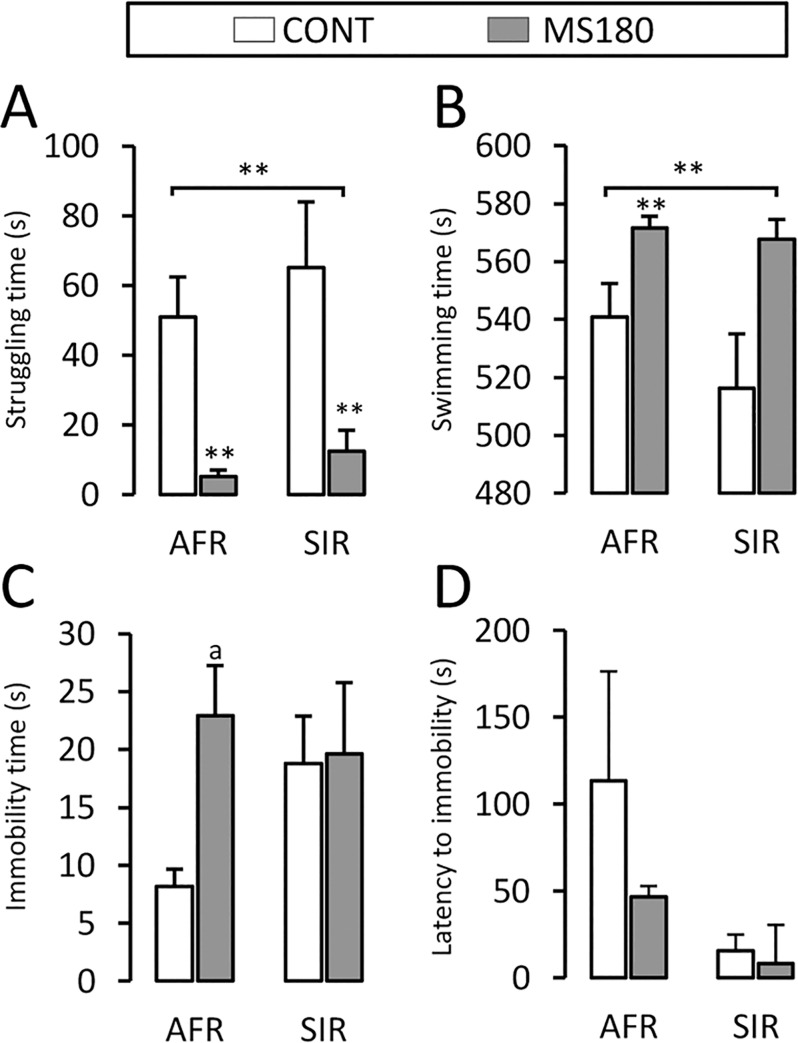
Maternal separation induces a passive coping strategy in the forced swimming test. A) Time spent struggling; B) time spent swimming; C) immobility time; and D) latency to immobility of maternally separated (MS180) and control (CONT) adult males reared under standard conditions (AFR) or social isolation (SIR). (Mean ± SEM, ANOVA *p ≤ 0.05, **p ≤ 0.01; Student’s *t*-test ^a^p ≤ 0.05; n = 7–8)

In the FST (Fig 4), two-way ANOVA (S1 Table) showed that MS180 alone (and therefore, not SIR) reduced struggling time (p ≤ 0.01) and increased swimming time (p ≤ 0.01), without any significant differences noted in the immobility time or latency to immobility. The effects of MS180 on immobility time were noted only as a trend (p ≤ 0.07).

**Fig 5:**
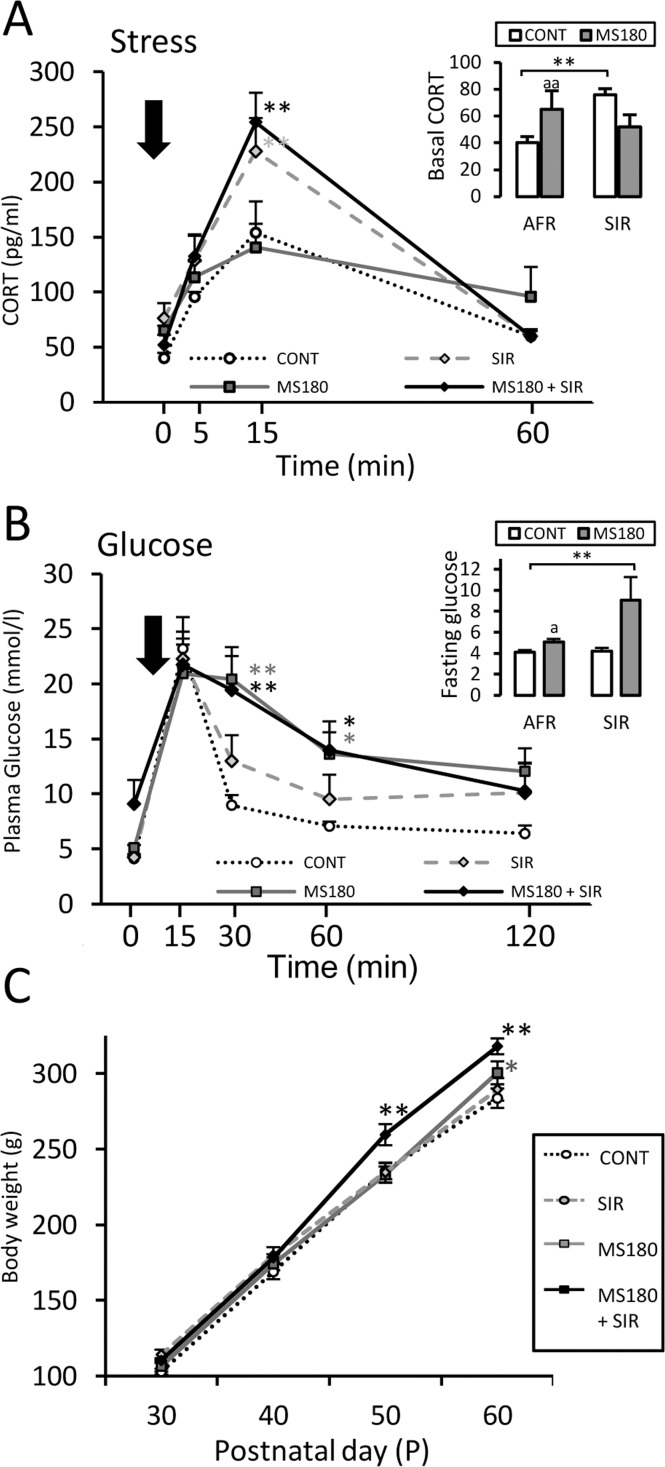
Effects of social isolation and maternal separation on stress and metabolic parameters. A) Corticosterone (CORT) plasma levels of maternally separated (MS180), control (CONT), socially isolated (SIR), and maternally separated and social isolation reared (MS180 + SIR) adult males before (inset) and at 5, 15, 30, and 60 min after stress exposure (2-min cold swim stress). B) plasma glucose levels in the IVGTT after a 12 h fast (inset) and 15, 30, 60, and 120 min after an intravenous glucose (50%, 1g/kg) load (Mean ± SEM, ANOVA *p ≤ 0.05, **p ≤ 0.01; Student’s *t*-test,^a^p ≤ 0.05, ^aa^p ≤ 0.01; n = 8). C) Body weight gain (Mean ± SEM, ANOVA*p ≤ 0.05, **p ≤ 0.01; n = 12)

SIR caused a significantly greater increase in plasma CORT levels 15 min post-stress in both control (SIR) and maternally separated (MS180 + SIR) animals, when compared to their AFR counterparts (Fig 5A). Three-way repeated measures ANOVA (S1 Table) showed significant effects of SIR (p ≤ 0.001), time after stress exposure (p < 0.0001), and interaction (p ≤ 0.001) on stress reactivity. However, no such effects were observed for MS180. Furthermore, SIR animals displayed a 90% increase in basal CORT levels when compared to their AFR control counterparts (p ≤ 0.01), and this effect was partially reversed in MS180 + SIR animals (Fig 5A, inset). Two-way ANOVA (S1 Table) of basal CORT indicated that neither MS180 nor the rearing conditions showed significant effects; however, a significant effect was noted because of their interaction (p ≤ 0.01). Similar to our observations for immobility time, the effects of MS180 on basal CORT were no longer observed in the ANOVA.

### SIR increased metabolic risk in MS180 rats

In the IVGTT (Fig 5B), glucose administration caused a significantly greater increase in blood glucose levels at 30 and 60 min post-injection in MS180 and MS180 + SIR animals, as compared to the AFR controls (p ≤ 0.01). Statistical analysis (S1 Table) revealed significant effects of MS180 (p = 0.003) and time after glucose administration (p < 0.0001); however, similar effects were not observed for rearing conditions. Furthermore, the glucose values of MS180 rats failed to be restored after 120 min (p ≤ 0.05 vs. basal). Two-way ANOVA (S1 Table) revealed significant effects of MS180 on fasting glucose (Fig 5B, inset, and Table 1; p ≤ 0.01); insulin (Table 1; p ≤ 0.01); triglycerides (Table 1; p ≤ 0.01); and total cholesterol (Table 1; p ≤ 0.05). Multiple comparisons showed that MS180 + SIR rats exhibited a 120% increase in fasting glucose levels (Fig 5B, inset, and Table 1;p ≤ 0.01) and a 65% increase in insulin (Table 1; p ≤ 0.05); however, the effects of MS180 on fasting glucose were only noted when single comparisons were performed (Student’s *t*-test, p ≤ 0.05). In addition, both MS180 and MS180 + SIR animals exhibited increased triglyceride and cholesterol levels (p ≤ 0.05). The QUICKY was affected in both MS180 and MS180 + SIR animals, regardless of the rearing conditions (p ≤ 0.05).

**Table 1:** Social isolation rearing induces metabolic syndrome in MS180 rats.

	CONT(n = 8)	SIR(n = 8)	MS180(n = 8)	MS180 + SIR(n = 8)
Metabolism				
Fasting corticosterone (ng/ml)	58.4 ± 8.6	94.0 ± 23.2	96.8 ± 22.0	58.7 ± 15.8
Fasting glucose (mmol/L)	4.1 ± 0.2	4.2 ± 0.2	5.1 ± 0.2	9.0 ± 2.1**
Insulin (pmol/L)	100 ± 24	93 ± 37	162 ± 30	166 ± 16*
QUICKY	0.39 ± 0.01	0.42 ± 0.02	0.34 ± 0.01	0.33 ± 0.01*
Triglycerides (mg/dL)	5.3 ± 0.3	6.7 ± 0.4	7.9 ± 0.6**	7.8 ± 0.9*
Total cholesterol (mg/dL)	130 ± 16	153 ± 6	211 ± 7**	180 ± 12**
Body weight at P60 (g)	285 ± 2.9	293 ± 8.3	304 ± 6.1*	323 ± 3.5**

(Mean ± SEM, ANOVA *p ≤ 0.05 **p ≤ 0.01)

### SIR augmented body weight gain in MS180 animals

We observed significant effects of MS180 (Fig 5C; p < 0.0001); SIR (p < 0.0001); age (p < 0.0001); and interaction between MS180 and age (p < 0.0001). MS180 and MS180 + SIR males displayed a 6.5% and 13% increase in body weight gain at P60, respectively (Table 1); however, this effect had an earlier onset in socially isolated animals, because multiple comparison analysis revealed an increase in body weight in MS180 + SIR animals from as early as P50 (Fig 5C).

### The effects of MS180 on coping strategy in the FST (but not on metabolic vulnerability) correlated with CORT levels

Pearson’s correlation analyses showed no association between basal or stress-induced CORT levels and FST behavior in control animals; however, we observed a significant CORT-immobility (p ≤ 0.01) negative association in SIR animals. Furthermore, MS180 animals showed positive CORT-swimming (p ≤ 0.01) and negative CORT-immobility (p ≤ 0.01) associations, whereas MS180 + SIR animals showed a positive association between CORT and struggling behavior (p ≤ 0.01).An ANCOVA model was used to test whether behavior parameters were associated with stress-induced CORT levels. A significant output of such an analysis establishes whether treatment effects persist, even after controlling for CORT levels. Statistical analysis for struggling behavior failed the homogeneity of regression coefficients test; therefore, ANCOVA was not performed. No significant effects of treatment were observed when behavior was controlled for stress-induced CORT levels. No correlation was observed between basal CORT and behavior; therefore, those data were not shown.

For metabolic risk parameters, correlation analysis showed significant values for fasting CORT-insulin association (p ≤ 0.01) in the control animals; however, this correlation was absent in the SIR, MS180, and MS180 + SIR groups (S1 Fig). A significant correlation between CORT and body weight was observed in MS180 animals (p ≤ 0.05). ANCOVA analysis showed that for fasting glucose, no significant effects of treatment were detected; however, significant effects were noted in insulin (F_3,30_ = 3.9, p ≤ 0.01); QUICKY (F_3,30_ = 6.4, p ≤ 0.01); and body weight (F_3,30_ = 4.6, p ≤ 0.01) when data were controlled for fasting CORT levels.

## Discussion

In the present study, we observed that maternal separation increases basal CORT levels and induces a passive coping strategy in the FST. MS180 was also associated with a mild increase in fasting glucose, insulin, total cholesterol, and triglyceride levels, as well as alterations in the IVGTT and QUICKY index. In addition, SIR significantly increased fasting glucose and body weight gain in MS180 rats, without further affecting behavior. Analyses of covariance indicated that MS180-induced effects on coping strategy and fasting glucose levels were related to HPA axis reactivity.

### Effects of MS180 on stress and metabolic risk

The effects of maternal separation on the HPA axis and coping strategy in the forced swimming test have been widely studied. As previously mentioned, MS180 animals displayed basal HPA axis hyperactivity and a passive coping strategy in the FST; however, stress reactivity remained unchanged. Slight variations in the separation procedures, such as the use of clean sawdust, removal of both litter and mother from the nest, and isolation from siblings during separation, could account for the wide variety observed in adult phenotypic outcomes of animals subjected to maternal separation [37]. Miyazaki and colleagues reported that rat pups singly isolated from the mother, presented a disturbance in synaptic delivery of AMPA receptors and barrel cortex plasticity; whereas pups separated from the mother, but kept among other pups showed no such disruption [38]. The programming of an adult fearful phenotype has been suggested to differ when pups are isolated from peers in a novel environment [39]. In the present study, we modified the separation procedure to increase the adversity experienced by both pups and dams [40], thus resulting in a more severe depressive-like phenotype that lacked the stress hyperreactivity of the procedures employed in previous studies.

Although the relationship between ELS and increased metabolic risk has been widely observed in humans, little effort has been made to determine a causal relationship in animal models. In adult rats, prenatal stress has been associated with low birth weight and altered glucose homeostasis [41,42]; however, the effects of postnatal stressors on metabolic vulnerability is not well understood. Only a few reports have focused on the effects of MS180 on glucose homeostasis in adults. In females, MS180 decreases insulin resistance biomarkers in chow-fed rats, but affects the response to an obesogenic diet later in life, and alters leptin mRNA levels [43]. Female rats are reportedly resistant to MS-induced effects [44,45]. This raises the question of whether sex differences could account for the differences observed in the present study. Furthermore, maternal separation has been suggested to decrease insulin levels and induce mild metabolic alterations in male Wistar rats [46]. In the present study, we demonstrated that in Sprague-Dawley animals, MS180 augments fasting glucose, insulin, cholesterol, and triglyceride levels and impairs glucose homeostasis in the IVGTT. ELS is known to interact with inborn stress reactivity and the genetic background of an individual to modify adult vulnerability [47–50]. Thus, these interactions could account for the disparities between the findings of our study and those of previous reports, particularly since low levels of maternal licking and grooming (a model of adverse early environmental influence) had limited effects on the behavior of Wistar offspring [51]. Moreover, MS180 has been shown to have little effect on stress sensitivity and anxiety in adult Wistar rats [52].

In the present study, MS180 animals exhibited a mild increase in fasting glucose levels, impaired glucose tolerance, and a slight increase in body weight and dyslipidemia. In type 2 diabetes, insulin receptors lose sensitivity to insulin, thereby rendering cells resistant to its effects. Consequently, circulating glucose levels become elevated. As a mechanism to cope with the elevated glucose levels, pancreatic cells respond by increasing their secretion of insulin. Furthermore, prenatal and early-life manipulations might induce metabolic alterations via epigenetic changes of various target gene systems [53], such as the dopaminergic circuitry or GC receptors, thereby effectively reprogramming neurobehavioral substrates related to feed intake/reward [54] and HPA axis feedback [55]. GC resistance due to an epigenetic downregulation of the GC receptor promoter has been proposed as a major mechanism for ELS-induced long-term programming [12,56]. Further analysis is required to determine whether a similar epigenetic mechanism could also account for MS180-induced insulin resistance.

### Effects of SIR on MS180 rats

In the present study, SIR increased basal CORT levels, and this effect was partially reversed in MS180 + SIR animals. No such effects were observed for MS180. Increased basal CORT levels in maternally separated animals were observed only when single comparisons were performed. These results are consistent with the mismatch hypothesis [51,57,58], which states that “adverse early-life experience triggers an adaptive process, thereby rendering an individual better adapted to stressful environments later in life” [51]. Nevertheless, the excessive CORT response observed in the present study in SIR and MS180 + SIR animals following exposure to stress might not entirely support the hypothesis. Moreover, no significant differences were observed between MS180 and MS180 + SIR animals among any of the other parameters, suggesting that SIR failed to promote adaptation in rats subjected to maternal separation. The effects of the relationship between MS180 and social isolation on HPA axis reactivity have been previously reported. Biggio et al. suggested that maternal separation attenuates the effect of social isolation on HPA axis responsiveness in adolescent rats [58]. These results are significantly different from those of the present study, because MS180 did not modify CORT levels in adolescent offspring, but induced partial recovery of reduced CORT levels caused by social isolation [58]. ELS has been observed to induce long-term alterations in an age-dependent manner, suggesting that the dissimilarities between our results and those previously described could be attributed to a differential effect of MS180 on adolescent and adult offspring. Although reports of the effects of social isolation on CORT levels differ [59–62], numerous studies indicate that this stressor causes an increase in the basal CORT of both males and females [63–66]. Moreover, consistent with our findings, MS180 has been observed to show increased HPA axis reactivity when combined with an unpredictable chronic stressor [67].

In agreement with previous studies [25,27,68], the present study showed that SIR has no effect on coping strategy in the FST. Furthermore, the behavioral scores of MS180 rats in the FST apparently were not adversely affected by SIR. ANCOVA analysis showed that treatment effects disappeared after controlling for CORT levels, suggesting a strong correlation between CORT and passive coping strategies in the FST. In addition, Pearson’s analysis showed a negative correlation effect. Therefore, these findings could support the hypothesis that ELS promotes depressive-like behavior through HPA axis programming, but not necessarily through increased CORT reactivity. Further analysis is necessary to characterize this relationship.

Previous studies have shown that MS180 might affect feed intake and body weight in AFR animals [31,69]. In contrast, SIR promotes feed intake and weight gain in MS180 rats [31], and causes sustained hyperphagia when MS180 animals are subjected to a fasting/re-feeding cycle. Based on our results, SIR increases body weight in MS180 animals from P50. As increased food consumption impacts anxiety-like behaviors in maternally separated animals, it has been proposed that HPA axis activity plays a crucial role in this enhanced vulnerability [32,69]. Elevated basal GC levels, such as those observed with SIR and MS180, can stimulate eating via interaction with appetite-regulating targets in the hypothalamus [70–72]. Moreover, in Sprague-Dawley rats and C57BL/6 mice, maternal separation and social isolation induce an upregulation of orexigenic neuropeptide Y in the hypothalamic region that is related to increased anxiety [73–76], and could lead to hyperphagia and consequently promote weight gain. However, this relationship has not been observed in maternally separated Wistar rats [77]. In addition, GCs enhance fat storage and contribute to visceral fat accumulation [72,78], and ELS effectively increases the deposition of abdominal fat [77]; however, this can be prevented by neonatal stimulation [79]. The lack of association between CORT levels and body weight observed in the present study suggest that SIR-induced body weight gain in MS180 animals could be related to both hyperphagia and increased peripheral fat deposition, but is not directly modulated by GC.

SIR induced various physiological alterations associated with MetS in MS180 rats, such as obesity (>10%), hyperglycemia (>100%), hyperinsulinemia (>60%), altered glucose homeostasis in the IVGTT, and dyslipidemia. In humans, MetS is defined as a cluster of glucose intolerance, hypertension, dyslipidemia, and central obesity, with insulin resistance as the key element in the pathogenesis [80]. Endothelial dysfunction, genetic susceptibility, elevated blood pressure, and chronic stress are several factors that also constitute to the syndrome [81]. In the present study, we observed that although maternal separation induced some alterations that indicated a mild metabolic imbalance (i.e., increased fasting glucose and altered QUICKY index), only MS180 + SIR animals displayed significant increases in body weight and hyperglycemia. Altogether, these results suggest that SIR induces MetS in MS180 rats. Further analysis is recommended to evaluate whether these conditions could be aggravated over a longer period (i.e., in aged animals) or in the presence of metabolic challenges.

### Coping strategy is related to circulating GC levels

In CONT animals, correlation analysis showed that there was no association between GC levels and coping strategy in the FST. However, we observed a significant association between fasting CORT levels and insulin, suggesting that under normal conditions, GC plays an important role in glucose homeostasis, but not on coping strategy. This correlation was modified by our treatments. Although both MS180 and SIR groups showed associations between stress-induced CORT and passive coping strategy in the FST, only body weight in the MS180 group was correlated with GC levels. Moreover, MS180 + SIR showed no correlation between CORT levels and any other parameter evaluated. Altogether, these results indicate that stress-induced effects on depressive-like behavior (but not metabolic imbalances) are related to circulating GC levels. In mammals, the actions of GC appear to have evolved to maintain and enhance energy stores for life-saving gluconeogenesis. They act on the brain to stimulate search behaviors, feeding of palatable foods, and emotionally relevant memories [82]. GCs are involved in multiple metabolic processes, including the regulation of insulin sensitivity and adipogenesis. Increased GC levels, such as those observed in Cushing’s syndrome, have been proposed as a central mechanism underlying the etiopathology of obesity and MetS. GC action partially depends on intracellular activation by 11β-hydroxysteroid dehydrogenase type 1 (11β-HSD1). This enzyme is expressed in several peripheral tissues, including the liver and adipose tissue, and is important for HPA axis activity. Prenatal stress has been shown to increase 11β-HSD1 in adipose tissue, and Morton et al. [83] reported that overexpression of 11β-HSD1 in adipose tissue results in visceral obesity and MetS in mice fed a high-fat diet. Adipose tissue expression of 11β-HSD1 and regulation of local cortisol levels might play a role in the development of obesity and metabolic disease [84], and could account for the disparities between circulating GCs and the effects of stress on metabolic risk.

## Conclusion

In the present study, we observed that ELS induces a passive coping strategy in the FST and increased HPA axis activity, causing mild metabolic alterations that could be aggravated when individuals are confronted with a socially adverse environment. As depressive-like behavior appeared without the onset of severe MetS in animal facility-reared animals, we believe that our findings support the hypothesis that depression and MetS vulnerability could be both consequences of ELS-induced deregulations, rather than consequences of each other. Correlation analysis showed that coping strategy in the FST (and not metabolic imbalance) is related to GC levels, suggesting that other mechanisms apart from GC-mediated effects could account for the effects observed.

## Supporting Information

S1 Fig Correlation Analysis. Linear regression analyses of coping strategy (immobility) and metabolic risk parameters plotted as corticosterone (CORT) dependent factors.
(TIF)

S1 Table ANOVA summary table. (DOCX)
