# Supplementary figures and images for: Early Life Stress Increases Metabolic Risk, HPA Axis Reactivity, and Depressive-Like Behavior When Combined with Postweaning Social Isolation in Rats
Source: PLoS One. 2016 Sep 9;11(9):e0162665. doi: 10.1371/journal.pone.0162665 (PMC5017766; doi:10.1371/journal.pone.0162665)

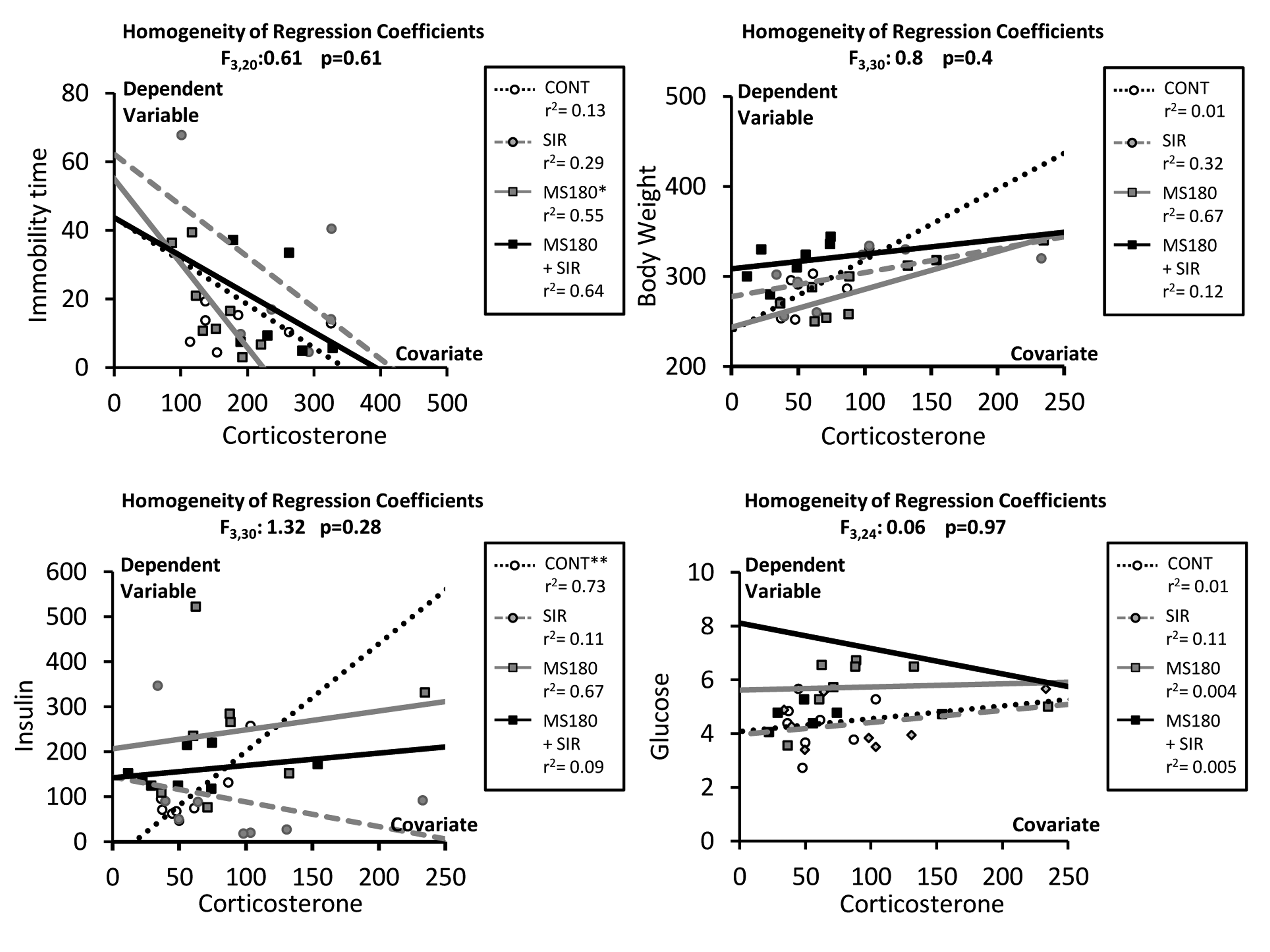

Supplement: S1 Fig — Linear regression analyses of coping strategy (immobility) and metabolic risk parameters plotted as corticosterone (CORT) dependent factors. (TIF) [file pone.0162665.s001.tif]
