# Supplementary material for: Early Life Stress Increases Metabolic Risk, HPA Axis Reactivity, and Depressive-Like Behavior When Combined with Postweaning Social Isolation in Rats
Source: PLoS One. 2016 Sep 9;11(9):e0162665. doi: 10.1371/journal.pone.0162665 (PMC5017766; doi:10.1371/journal.pone.0162665)
Supplement: S1 Table — (DOCX) [file pone.0162665.s002.docx]

| **ANOVA SUMMARY TABLE** | | | | | | | | | | | |
| --- | --- | --- | --- | --- | --- | --- | --- | --- | --- | --- | --- |
|  | | **n** | **FACT A: MS180** | | **FACT B: SIR** | | | | **A X B** | | |
| **STRESS REACTIVITY** | | | | | | | | | | | |
| BASAL CORTICOSTERONE | 8 | | ns | | | ns | | F_1,31_:4.0, p = 0.009 | | | |
|  |  | | **A: MS180** | **B: SIR** | | **C: TIME** | **AXB** | **AXC** | | **BXC** | |
| STRESS RESPONSE | 8 | | ns | F_1,127_:7.6, p = 0.001 | | F_1,127_:52, p < 0.0001 | ns | ns | | F_1,127_: 7.6, p = 0.001 | |
| **FST** |  | |  | | |  | |  | | | |
| STRUGGLING | 7-8 | | F_1,30_:17.2, p = 0.003 | | | ns | | ns | | | |
| SWIMMING |  | | F_1,30_:11.7, p = 0.002 | | | ns | | ns | | | |
| IMMOBILITY |  | | ns (F_1,30_:3.5, p = 0.07) | | | ns | | ns | | | |
| LATENCY TO IMMOBILITY |  | | ns | | | ns (F_1,30_:3.7, p = 0.06) | | ns | | | |
| **METABOLISM** |  | |  | | |  | |  | | | |
| FASTING CORTICOSTERONE | 8 | | ns | | | ns | | F_1,31_:4.0, p = 0.05 | | | |
| GLUCOSE | 8 | | F_1,31_: 7.0 P = 0.01 | | | ns | | ns | | | |
|  |  | | **A: MS180** | **B: SIR** | | **C: TIME** | **AXB** | **AXC** | | | **BXC** |
| IVGTT | 8 | | F_1,140_:10.5, p = 0.003 | ns | | F_1,140_:38.3, p < 0.0001 | ns | F_1,140_:3.5 p < 0.009 | | | ns |
| INSULIN | 8 | | F_1,31_: 5.6 p = 0.02 | | | ns | | ns | | | |
| QUIKY | 8 | | F_1,31_: 13.3 P = 0.001 | | | ns | | ns | | | |
| TRIGLYCERIDES | 8 | | F_1,31_: 8.2, p = 0.007 | | | ns | | ns | | | |
| TOTAL CHOLESTEROL | 8 | | F_1,31_: 4.6, p = 0.03 | | | ns | | ns | | | |
| **WEIGHT** |  | |  | | |  | |  | | | |
| BODY WEIGHT (P60) | 8 | | F_1,31_: 18.9, p = 0.002 | | | F_1,31_: 5.6, p = 0.02 | | ns | | | |
|  |  | | **A: MS180** | **B: SIR** | | **C: AGE** | **AXB** | **AXC** | | | **BXC** |
| WEIGHT GAIN | 12-16 | | F_1,256_:15.2, p < 0.0001 | F_1,256_:596, p < 0.0001 | | F_1,256_:2514, p < 0.0001 | ns | F_1,256_:9.4, p < 0.0001 | | | ns |
